# Successful conservative management of advanced pyogenic sternoclavicular joint arthritis with osteomyelitis and pulmonary infiltration: a case report

**DOI:** 10.1186/s13256-024-04684-z

**Published:** 2024-08-26

**Authors:** Takahito Sugihara, Yoshifumi Sano, Takashi Ueki, Takao Ishimura, Masashi Takeda, Yosuke Kiriyama, Yu Mori, Nobuhiko Sakao, Shinji Otani, Hironori Izutani

**Affiliations:** Department of Cardiovascular and Thoracic Surgery, Ehime University Graduate School of Medicine, 454, Shitsukawa, Toon, Ehime 791-0204 Japan

**Keywords:** Case report, Antibiotic therapy, Conservative management, Methicillin-sensitive *Staphylococcus aureus*, Sternoclavicular joint arthritis

## Abstract

**Background:**

Sternoclavicular joint arthritis is a rare condition that poses considerable diagnostic and therapeutic challenges, leading to severe complications and a high mortality rate. Although surgical interventions are often considered necessary for advanced cases, some reports have suggested that conservative management with antibiotic therapy can be effective in certain cases. However, to our knowledge, there are no reports of successful conservative treatment in cases exhibiting aggressive spread. This report highlights a case of advanced sternoclavicular joint arthritis with bone destruction and pulmonary infiltration, successfully treated conservatively with outpatient antibiotic therapy.

**Case presentation:**

A 58-year-old Japanese male presented with a 1-month history of left-sided shoulder pain. Contrast-enhanced computed tomography showed abscess formation and clavicular bone destruction, with infiltrative shadows suggesting lung involvement. The diagnosis of sternoclavicular joint arthritis was made, and outpatient oral antibiotic therapy was initiated. The patient exhibited a marked reduction in inflammatory marker levels and symptoms, and antibiotic therapy was discontinued after 3 weeks, with no recurrence observed at a 4-month follow-up.

**Conclusions:**

This case highlights that conservative management with antibiotics can be effective for treating advanced sternoclavicular joint arthritis, emphasizing the need for individualized management and further research into non-surgical treatment options.

## Background

Sternoclavicular joint (SCJ) arthritis is a rare condition requiring prompt and accurate diagnosis and treatment, as delayed diagnosis can result in serious complications, such as osteomyelitis, mediastinitis, and sepsis, which are associated with a high mortality rate of 10% [1]. The diagnostic criteria for SCJ arthritis are lacking [2], and its diagnosis is typically based on a thorough history, physical examination, laboratory workup, imaging findings, and arthrocentesis. Although surgical procedures are considered inevitable, conservative management is effective in some cases [3, 4]. However, previous reports were limited to patients with mild inflammation. Herein, we report a case of successful conservative outpatient treatment of advanced SCJ arthritis with bone destruction and pulmonary infiltration.

## Case presentation

A 58-year-old Japanese male with a history of chronic sinusitis, hyperuricemia, and depression presented with left-sided shoulder pain persisting for 1 month. He was not a smoker and only drank socially. He had no remarkable family medical history, and he worked in the hospitality industry. He had no history of medication use. Computed tomography (CT) performed before presentation revealed a low-density area around the left SCJ, prompting referral to our institution.

Upon examination, the patient was hemodynamically stable, with a body temperature of 36.9 ℃, blood pressure of 133/86 mmHg, and a pulse rate of 78 beats per minute (bpm). Physical examination revealed mild swelling and tenderness around the left SCJ. In addition, no skin breakdown or indwelling prosthetic devices including intravascular catheters and cardiac devices were present. No evidence of dental caries, swollen tonsils, or significant limitation of left-shoulder movement was observed. Laboratory findings revealed a white blood cell (WBC) count of 11,300 cells/mm^3^ and a C-reactive protein (CRP) level of 5.16 mg/dL (Table 1). Moreover, the levels of tumor markers, including carcinoembryonic antigen, cytokeratin 19 fragment, and squamous cell carcinoma antigen, were within normal ranges.

**Table 1:** Laboratory findings on presentation

**Hematology**		**Units**
WBC	11,300	/µL
Neu	82.4	%
Lym	11.4	%
Mon	3.6	%
Eos	2.1	%
Bas	0.5	%
RBC	501 × 10^4^	/µL
Hb	14.3	g/dL
Ht	44.1	%
Plt	34.2	/µL
**Biochemistry**		
TP	8.2	g/dL
Alb	3.6	g/dL
AST	28	U/L
ALT	30	U/L
LDH	165	U/L
ALP	122	U/L
BUN	11	mg/dL
Cre	0.83	mg/dL
Na	141	mmol/L
K	4.6	mmol/L
Cl	101	mmol/L
Ca	9.3	mmol/L
HbA1c	6.9	%
CRP	5.16	mg/dL
**Serology**		
Procalcitonin	0.151	ng/mL
β-D-glucan	9.3	pg/mL

WBC, white blood cell; Neu, neutrocyte; Lym lymphocyte; Mon, monocyte; Eos, eosinophils; Bas basophils; RBC, red blood cell; Hb, hemoglobin; Ht, hematocrit; Plt, platelet; TP, total protein; Alb albumin; AST, aspartate aminotransferase; ALT, alanine aminotransferase; LDH, lactate dehydrogenase; ALP, alkaline phosphatase; BUN, blood urea nitrogen; Cre, creatinine; Na, sodium; K, potassium; Cl, chloride; Ca, calcium; HbA1c, hemoglobin A1c; CRP, C-reactive protein

**Fig. 1:**
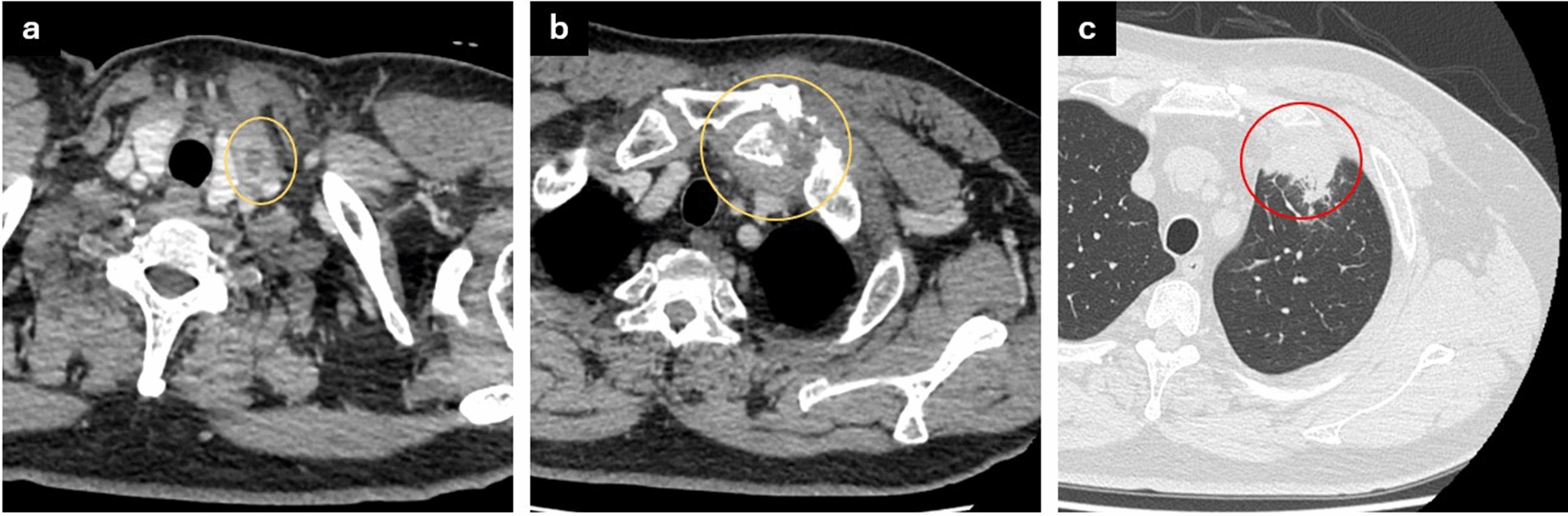
Contrast-enhanced computed tomography findings at the initial presentation. **a** Neck abscess (yellow circle), **b** soft-tissue swelling around the left sternoclavicular joint with clavicular bone destruction (yellow circle), and **c** infiltrative shadows in the left lung apex suggesting the spread of inflammation to the lung (red circle)

Contrast-enhanced CT revealed a low-density area extending from the anterior-neck muscles to the posterior sternal region and left clavicle with mild peripheral enhancement, indicating abscess formation, and clavicular bone destruction (Fig. 1a, b). Furthermore, infiltrative shadows in the left lung apex suggested the spread of inflammation (Fig. 1c). Neck ultrasonography revealed a hypoechoic area within the muscles of the anterior neck, suggestive of abscess formation.

The patient was diagnosed with SCJ arthritis, and oral sulbactam/ampicillin (1125 mg/day) was initiated in an outpatient setting to provide coverage against anaerobic germs. Additionally, ultrasound-guided aspiration of the cervical abscess was performed, and methicillin-sensitive *Staphylococcus aureus* was detected in the aspirate culture, supporting the continuation of oral antibiotics. Cellular cytology revealed no malignant findings. Weekly follow-ups were conducted, including blood tests, CT, and neck ultrasonography. A total of 1 week after starting treatment, pain, swelling, and inflammatory marker values such as WBC counts and CRP levels showed improvement on examination. The antibiotic was switched to oral levofloxacin (500 mg/day), which showed higher sensitivity on blood culture tests.

**Fig. 2:**
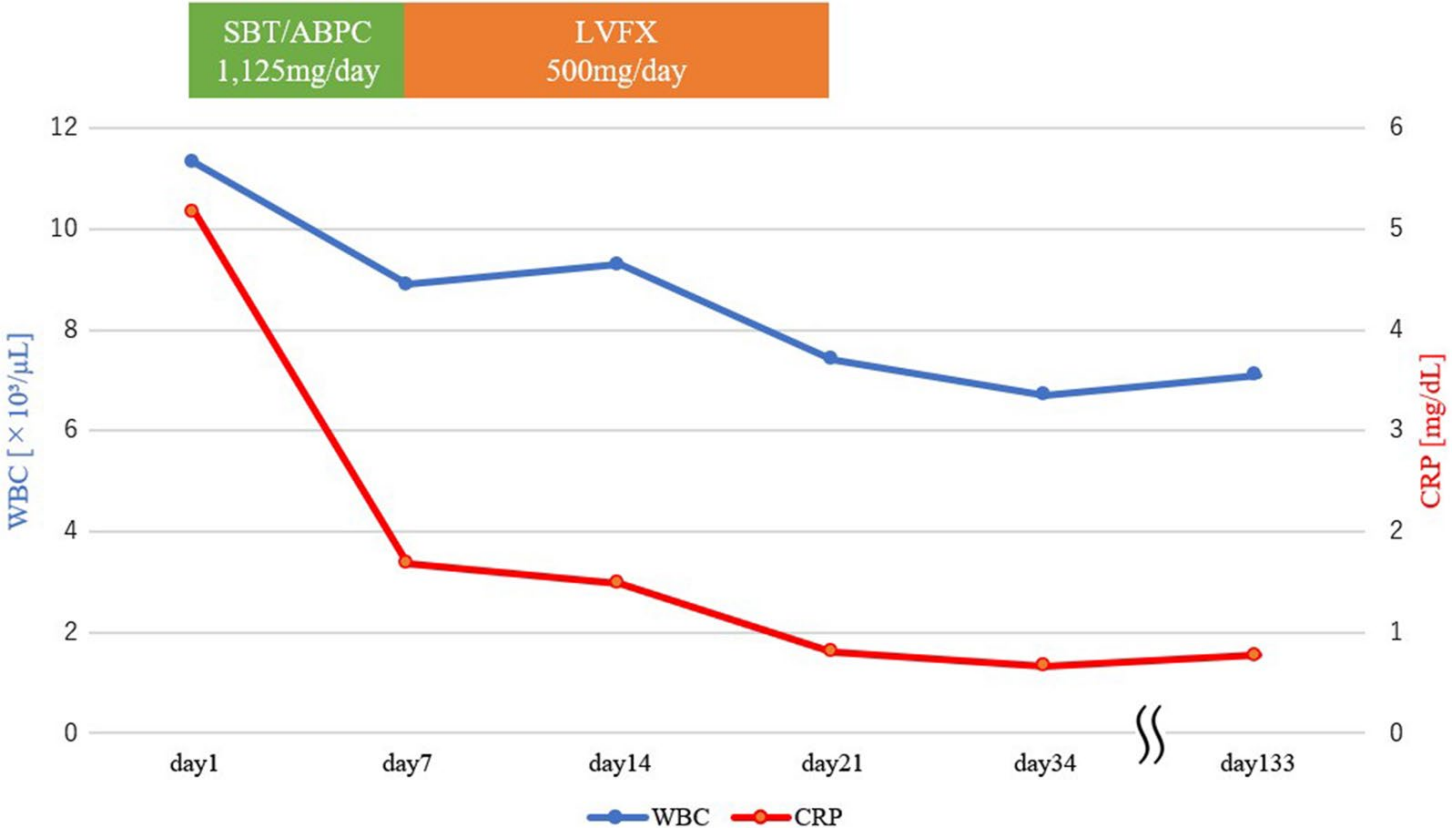
Therapeutic course of sternoclavicular joint arthritis. CRP, C-reactive protein; LVFX, levofloxacin; SBT/ABPC, sulbactam/ampicillin; WBC, white blood cell

**Fig. 3:**
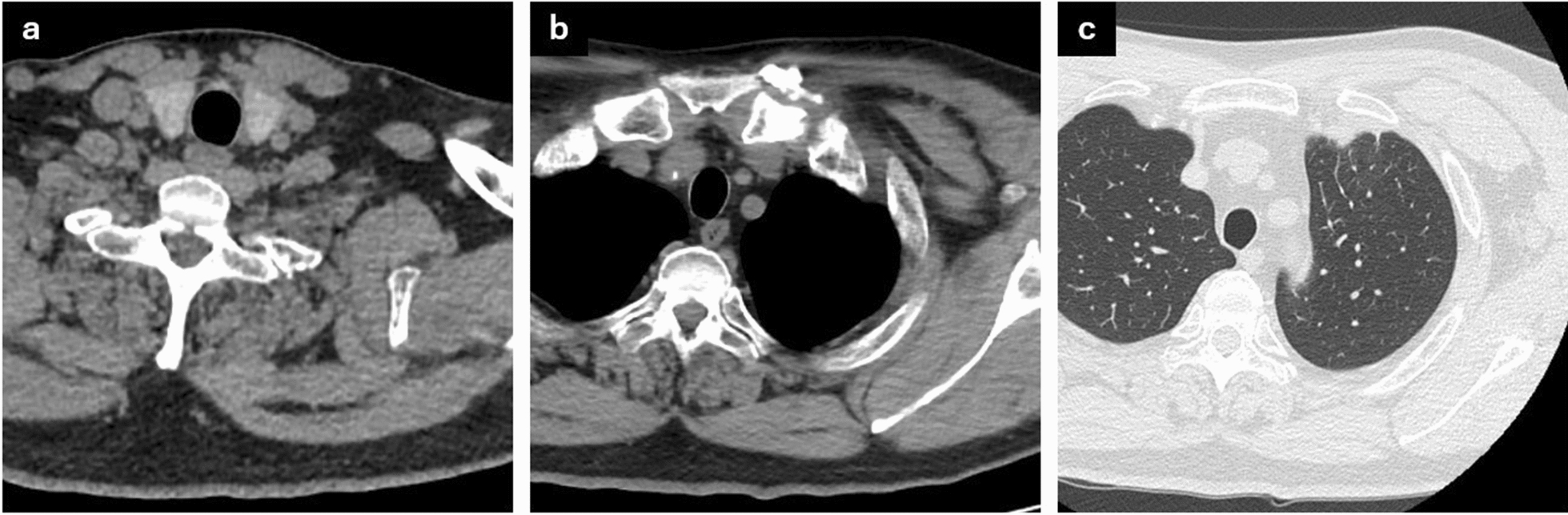
Computed tomography 4 months after completing antibiotic treatment. **a** Complete resolution of the neck abscess and significant improvement in **b** the soft-tissue swelling around the left sternoclavicular joint and **c** lung inflammation

A total of 3 weeks after treatment initiation, the inflammatory marker levels normalized, and antibiotic therapy was discontinued (Fig. 2). No symptom recurrence was observed at the 4-month follow-up after completing antibiotic treatment (Fig. 3). Additionally, the patient had no difficulty with upper limb movement and experienced no residual swelling or pain.

## Discussion and conclusions

This report presents new findings that conservative treatment with antibiotics and other measures can lead to improvement even in cases of SCJ arthritis with abscess formation and bone destruction, which were previously thought to necessitate surgical intervention. The basis of this approach lies in the advancements in medicine, including antibiotics and other drugs, and the idea that these advancements make new methods, rather than traditional ones, sometimes effective.

The estimated prevalence of SCJ infections is less than 1% of all septic arthritis cases [5]. Ross *et al*. [6] reported 180 cases of SCJ infections, almost half of which were caused by *S. aureus*, followed by *Pseudomonas aeruginosa* (10%), and *Brucella melitensis* (7%). Clinical symptoms included pain around the SCJ (78%), fever (65%), and shoulder-joint pain (24%). Risk factors included frequent use of intravenous drugs (21%), spread of infection from a distant site (15%), diabetes (13%), trauma (12%), and central-venous-line infection (9%). However, 23% of cases occurred in healthy individuals without any identified risk factors [6]. Although standard diagnostic criteria have not been established, CT-guided arthrocentesis yields a positive culture in over 50% of cases [7].

Conventionally, SCJ arthritis management includes surgery, ranging from simple incision and drainage to extensive debridement and reconstruction [8, 9]. Surgical resection combined with muscle transposition provides effective long-term outcomes. Joint resection combined with intravenous antibiotics effectively and expeditiously eliminates the disease. In particular, *en bloc* joint resection and bone and soft-tissue debridement are indicated in the case of extensive bony destruction, chest-wall phlegmon or abscess, retrosternal abscess, mediastinitis, or pleural extension [10]. Although surgical intervention combined with targeted antibiotic therapy is the mainstay of treatment, several cases of SCJ arthritis without life-threatening complications have been successfully treated with antibiotic therapy alone [3, 6, 11]. Surgical intervention is associated with morbidity risks not encountered with antibiotic therapy. Factors such as adverse reactions to anesthesia, postsurgical infections, and the potential need for additional surgeries must be carefully considered before surgical treatment. Furthermore, the instability of the SCJ induced by surgical intervention may lead to impairments in upper limb elevation [11]. Notably, for patients without severe signs of osteomyelitis or systemic infection, treatment with antibiotics alone should be considered a viable option [2]. Successful medical management has been reported even in patients with clavicular osteomyelitis. For example, a patient showing early signs of osteomyelitis was successfully treated with a 4-week course of intravenous antibiotics [12].

In our patient, outpatient antibiotic therapy was selected on the basis of the mild elevation of inflammatory marker levels and absence of high fever despite evidence of inflammatory spread. This case emphasizes substantial improvement in antibiotics and advancements in diagnostic equipment and technologies.

In conclusion, we encountered a rare case of SCJ arthritis with pulmonary extension and osteomyelitis that was successfully treated with antibiotic therapy in the outpatient setting. This report demonstrates that conservative treatment may be effective even in appropriately selected cases of advanced SCJ arthritis.

## Data Availability

Further information about this study is available by contacting the corresponding author.

## Abbreviations

CRP: C-reactive protein
CT: Computed tomography
SCJ: Sternoclavicular joint
WBC: White blood cell
